# Anticoagulation Therapy for Non-valvular Atrial Fibrillation: A Mini-Review

**DOI:** 10.3389/fmed.2020.00350

**Published:** 2020-07-21

**Authors:** Jia Wu, Yonggang Zhang, Xiaoyang Liao, Yi Lei

**Affiliations:** ^1^Department of International Medical Center/Ward of General Practice, West China Hospital, Sichuan University, Chengdu, China; ^2^Department of Periodical Press, National Clinical Research Center for Geriatrics, West China Hospital, Sichuan University, Chengdu, China

**Keywords:** atrial fibrillation, anticoagulation, non-valvular heart disease, direct-acting oral anticoagulant, clinical trial

## Abstract

Anticoagulation therapy is an important method of preventing stroke in individuals with atrial fibrillation (AF). Atrial fibrillation is a quivering or irregular heartbeat that can lead to blood clots, stroke, heart failure, and other heart-related complications. Clinical guidelines on AF consistently recommend long-term oral warfarin to treat valvular atrial fibrillation (VAF). However, due to varying risks of blood clots and stroke associated with different types of non-valvular atrial fibrillation NVAF, it is unclear whether direct oral anticoagulant (DOAC) can replace warfarin. Despite a recent increase in evidence on the effectiveness and the importance of anticoagulant therapy in preventing thromboembolic events associated with NVAF, clinical prevention strategies remain complex. Given the complexities associated with clinical use of anticoagulants for patients with NVAF, this review aims to offer guidance on patient anticoagulant use based on current available evidence.

## Introduction

Atrial fibrillation (AF) is a common type of arrhythmia. There are currently 335 million individuals with AF worldwide (1), with an overall prevalence rate of 2.9% (2). With an aging global population and changing lifestyles, the incidence of AF is increasing rapidly. The prevalence of AF is around 0.1% for individuals under 55 years old, more than 5% in people over 65 years old, and more than 9% in people over 80 years old (3).

The main negative effects of AF are thrombosis and embolism. For example, the incidence of embolic events in patients with non-valvular atrial fibrillation (NVAF) is 5% per year, which accounts for 15–20% of all cerebral embolism events (4). These consequences of stroke could increase the risks of death and disability by more than 5-fold (5, 6). In general, the fatality rates for stroke are 15, 25, and 50% in the 1-month, 1-year, and 5-years post-stroke periods, respectively (7). However, patients with stroke caused by AF experience persistent recurrences for 5 years as well as higher early mortality rates (7). Therefore, clinical guidelines have identified anticoagulation for individuals with NVAF, as the cornerstone approach to controlling ischemic stroke. However, since clinical risks of atrial fibrillation increase with age, more proactive prevention methods are needed for older individuals.

Over the past 50 years, clinical guidelines have recommended the use of oral anticoagulant (OAC) in NVAF, from the most widely used warfarin to the more effective direct acting oral anticoagulants (DOAC) (8). Most data have shown that the use of OACs in NVAF can reduce the risk of stroke. Studies have shown that anticoagulation therapies can decrease the incidence of stroke by 50% and prevent the recurrence of stroke (9–11). According to data extracted from electronic medical records over the last 10 years in the UK, a 1% increase in anticoagulant use can result in 0.8% decrease in the incidence of stroke associated with AF (12).

In 2010, the Food and Drug Administration (FDA) approved the first DOAC for stroke prevention in AF, dabigatran. Since then, the FDA has approved other DOACs including rivaroxaban in July 2011, apixaban in December 2012, and edoxaban in January 2015. Although several DOACs have become available in the last 10 years, a Phase III trial of more than 100,000 subjects found that the various DOACs have similar efficacy in preventing stroke in patients with NVAF (13–16).

By 2016, DOAC prescriptions exceeded warfarin prescriptions for patients with AF (13). As the use of DOACs has increased, more data have become available on their efficacy for NVAF, as well as on their safety for patients. In 2019, AF clinical guidelines from Europe and the United States prioritized the use of DOACs over vitamin K antagonists (VKAs) for NVAF therapy in most situations (17, 18). However, there are risks associated with these drug use, including potential gastrointestinal bleeding and fatal intracranial hemorrhage. Such side effects can lead to insufficient implementations of prevention strategies. Given the challenges facing the selection of anticoagulants in patients with NVAF, we have summarized the differences in mechanism of action between traditional VKAs and DOACs based on a review of recent evidence and clinical use strategies for different individuals.

## Mechanism of Action of VKAs and DOACs

**Figure 1:**
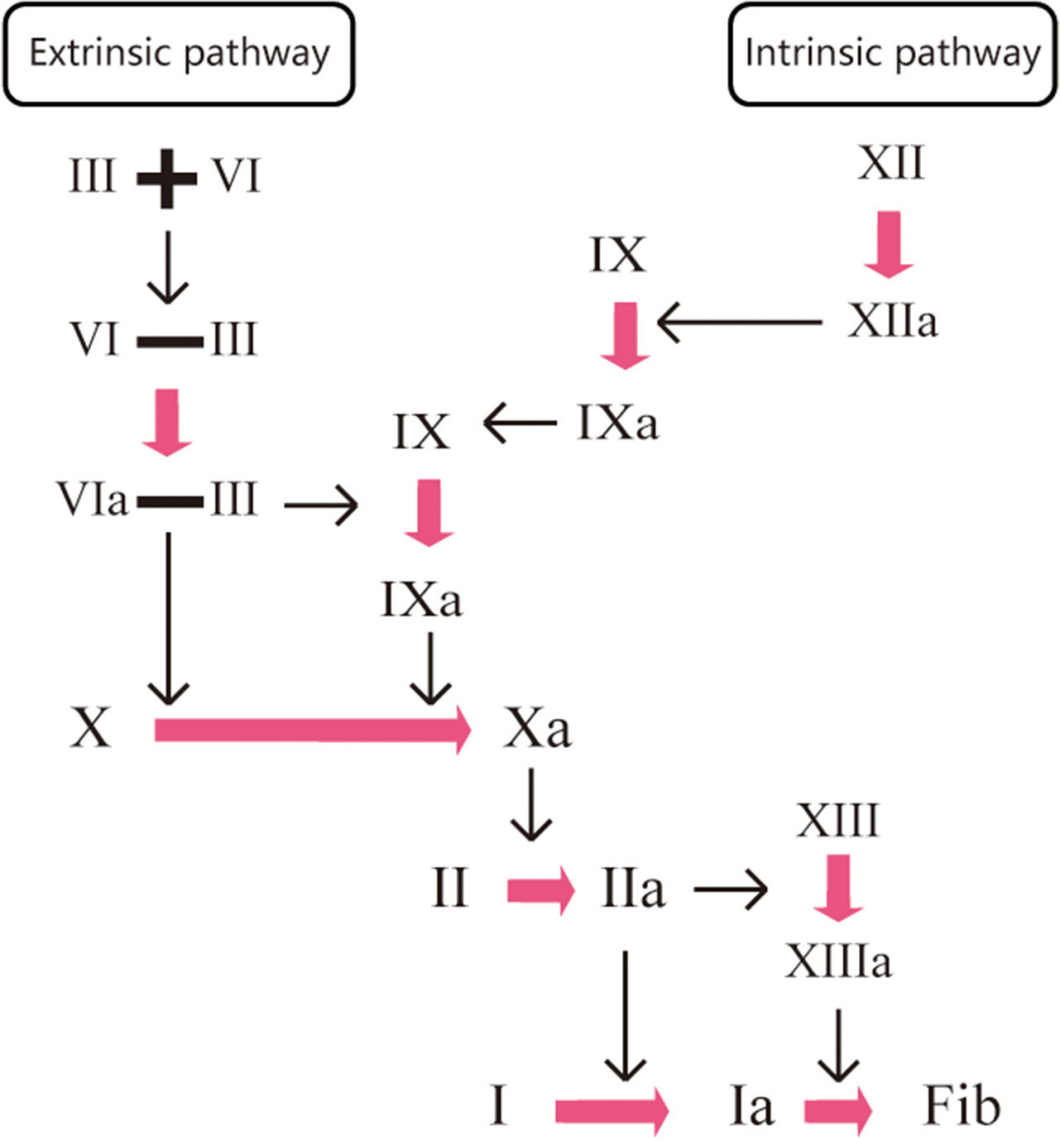
Mechanism of anticoagulant action.

Under normal conditions, the clotting process of the human body is a waterfall-like enzymatic cascade reaction (19). The main principle of anticoagulant drugs is to block the cascade reaction by directly or indirectly inhibiting one or more condensation factors in the coagulation process, thus preventing the development of thrombosis. VKAs induce anticoagulant action by non-specific indirect inhibitions of clotting factors (factors X, IX, IX, IX, VII, and II). Warfarin, a VKA, is a coumarin-derived, multi-target and non-selective oral anticoagulant that relies on vitamin K. It acts on the coagulation factors (VII, IX, and X) at the early stage of the coagulation cascade response to inhibit thrombin production and factor II activation. However, it does not affect the protein synthesis of coagulation factors, instead acting by inhibiting their carboxylation process. Therefore, the process has no effect on coagulation factors that have already been activated in the body. DOACs, due to their high specificity, induce anticoagulants by directly blocking the activities of coagulation factors Xa and IIa cells. An example of a DOAC is the IIa inhibitor dabigatran, which acts on the last step of the coagulation cascade response. Dabigatran directly inactivates the thrombin that has been produced (IIa), exerting anticoagulant effects by blocking fibrinogen cleavage to fibrin. The factor Xa inhibitors, such as rivaroxaban, apixaban, and edoxaban, act on the common pathway of endogenous and exogenous coagulation reactions. The inactivation of one Xa inhibitor can result in the reduction of 1000 IIa cells, which effectively inhibits the production of thrombin (IIa) and achieves anticoagulant effects (Figure 1).

## Potential Problems in Anticoagulant Therapy With VKAs in Real-World Observational Studies

In the past half century, warfarin has been used in thrombosis, atrial fibrillation, artificial valve replacement and other indications (20). A meta-analysis of five studies established the effectiveness and safety of warfarin anticoagulant therapy (21–23). Moreover, a meta-analysis found that warfarin reduced the overall stroke risk of patients with AF by 68% and all-cause mortality by 33% (24).

However, systematic reviews of real-world data have shown that there are limitations to the use of warfarin in clinical practice due to its narrow therapeutic window and poor quality of anticoagulation control. Time to treatment (TTR), the proportion of time that the patient's INR between 2 and 3, is the standard method for assessing the quality of anticoagulation control and the risk-benefit profile of warfarin. Potential interactions between warfarin and certain foods or medications can affect INR levels, if the criteria for TTR are not met, there will be high risks of embolism or bleeding (25), thus restricting the use of the medicine in clinical practice. Furthermore, NVAF comorbid conditions, such as other heart disease, liver disease, renal insufficiency, and infections can cause instability of INR results and increase the risk of bleeding. Some foods and drugs interact with warfarin (26, 27), affecting INR levels, so diet and use of other medications are restricted while taking warfarin. Frequent blood draws are needed to detect INR, and this can result in decreased patient compliance. Patient non-compliance, including self-termination of treatment, is a major factor leading to adverse events in individuals treated with warfarin. Due to the risk factors mentioned above, warfarin is not an ideal anticoagulant therapy for stroke prevention and there continues to be a need for improved long-term anticoagulant treatment.

## A Comparison of DOACs and VKAs

There is substantial clinical experience with use of DOACs, and patient-reported outcomes have been summarized in a systematic review (28). In addition, clinical trials have found that the newer oral anticoagulants can reduce the stroke rate by 19% compared with warfarin (11, 29). Based on the available evidence, DOACs have been recommended in national guidelines on the prevention of stroke for individuals with NVAF and who have one or more risk factors for stroke. However, in part due to the large sample size required to detect differences between groups in drug trials, there are currently no head-to-head, blinded studies comparing VKAs and DOACs (30).

Real-world observational studies of DOACs have been conducted, particularly on dabigatran, which was the first DOAC on the market (31). Observational studies have validated the results of the 2009 RE-LY trial evaluating dabigatran (15). RELY-ABLE (32) was a follow-up to the RE-LY trial and addressed the long-term effectiveness and safety of 150 and 110 mg doses of dabigatran. The RELY-ABLE trial found that annual rate of stroke or systemic embolism was similar in the two groups; 1.46% per year in the 150 mg group and 1.60% per year in the 110 mg group (HR: 0.91, 95% CI: 0.69–1.20). However, individuals in the 150 mg group had significantly more bleeding events than those in the 110 mg group (HR: 1.26, 95% CI: 1.04–1.53), suggesting that bleeding should be carefully observed in patients receiving high-dose dabigatran.

The results of the 2017 RE-CIRCUIT (33) study showed that patients who underwent catheter ablation had a lower probability of clinically significant bleeding and severe side effects with dabigatran than with warfarin (34). The RE-DUALPCI trial (35) found that, in patients with AF undergoing percutaneous coronary intervention–associated stent placement, the incidence of hemorrhage and clinically-related non-hemorrhage in the dabigatran group was significantly lower than in the warfarin triple therapy group at 3 years. The annual incidence of hemorrhage associated with long-term anticoagulant therapy was 1.1–8.1%. The most common sites of bleeding were the skin, mucous membranes, gastrointestinal tract, and genitourinary tract. Intracranial hemorrhage (ICH) is the most serious and can endanger patients' lives. The primary endpoint, risk of bleeding, was reduced by 48 and 28% in the dual therapy dabigatran 110 and 150 mg groups, respectively, compared with the warfarin triple therapy group (36). Clinical trials ROCKET-AF (37), ARISTOLE (38), ENGAGE AF TIMI-48 (39) also showed that DOACs (rivaroxaban, apixaban, edoxaban) is more effective than warfarin, edoxaban gastrointestinal bleeding in the high dose group (60 mg/d) was higher compare with warfarin (see Table 1). In a systematic review of including 170,814 patients treated with apixaban, the majority results showed that warfarin was associated with a lower risk of stroke and systemic embolic events, as well as major bleeding, particularly ICH (46%RRR; p <0.00001) (40). Rutherford et al. (41) recently published a large-scale observational study from Norway, treatment with DOACs for 65,563 AF patients firstly, the results found no statistically significant difference in stroke or SE risk between dabigatran group, rivaroxaban or apixaban. The risk of dabigatran and apixaban was significantly reduced major bleeding compared to rivaroxaban.

**Table 1:** Clinical Trials of NVAF for DOACs.

**Study**	**Drug**	**Outcome**
RE-LY (15)	Dabigatran	150 mg efficacy of group was better than warfarin, and the relative risk was reduced by 34%, and the 110 mg effect was not inferior to warfarin.
RELY-ABLE (32)	Dabigatran	Embolism in the 150 mg group: 1.46% per year; 110 mg group: 1.60% per year
RE-CIRCUIT^*^ (33)	Dabigatran	A lower probability of clinically significant bleeding and severe side effects
RE-DUALPCI^#^ (34)	Dabigatran	Hemorrhage and clinically-related non-hemorrhage was significantly lower
ROCKET-AF (37)	Rivaroxaban	75-years-old elderly patients have better efficacy and safety
ARISTOLE (38)	Apixaban	Stroke and body circulation embolism decreased by 21%
ENGAGE AF TIMI-48 (39)	Edoxaban	Gastrointestinal bleeding in the high dose group (60 mg/d) was higher

* *RE-CIRCUIT: patients with underwent catheter ablation*.

# *RE-DUALPCI: patients with undergoing percutaneous coronary intervention–associated stent placement*.

According to the latest European guidelines, patients with AF with a CHA_2_DS_2_-VASc score >2 are advised to use DOACs or VKAs to reduce the risk of stroke. However, for patients with CHA_2_DS_2_-VASc of 1, the risk of thromboembolic is only 0.6–1.3%, but the risk of bleeding will increase, which should be determined according to the individual balance between thromboembolism and bleeding risk. The preference of treatment decision should be to do beneficial, not harmful to patients, not only to avoid stroke. Based on this, it is a key prerequisite to start OAC to evaluate the risk of major bleeding of patients. For patients with CHA_2_DS_2_-VAS_C_ of 1, if it has been decided to start OAC, DOAC with higher clinical net benefit should be preferred instead of VKA (42, 43).

Because NVAF patients tend to have multiple comorbidities, clinical trials cannot ignore real-world safety studies of warfarin's use in specific high-risk populations.

Numerous studies have shown that increasing age is an independent risk factor for NVAF, leading to increased morbidity and mortality (44). In individuals over 80 years old with NVAF, the mortality rate is 9% (7). In addition, older patients with AF have higher rates of thrombosis and bleeding than younger patients. Several real-world observational studies have included analyses stratified by age, some with a highest age group of 80 years old (45–47). Despite the higher risk of bleeding in older individuals, a Japanese cohort study found that warfarin had a positive net clinical benefit in individuals ≥90 years due a reduced risk of ischemic stroke (48). Research reviews have generally found more favorable outcomes for DOACs than warfarin in the oldest patients, thus DOACs may still be recommended for elderly patients, despite the risks.

About 10–15% of AF patients have chronic kidney disease (CKD), and severe renal insufficiency is an independent risk factor for stroke in AF patients. Given that risks of stroke and hemorrhage are higher in AF patients with renal insufficiency, the selection of oral anticoagulants should be made more carefully for these patients. Warfarin is one of the most commonly used anticoagulants in patients with CKD (49). Clinical practice guidelines consistently recommend warfarin for anticoagulation in AF patients with CKD or end-stage renal disease (ESRD) (50). However, studies on the safety and effectiveness of warfarin in AF patients with CKD have found that, compared with healthy populations, warfarin does not reduce the incidence of ischemic stroke and it increases the risk of intracranial hemorrhage (3 vs. 1% per year). Therefore, it is uncertain whether warfarin can be used as an anticoagulant in patients with severe CKD or ESRD.

In patients with renal insufficiency, clinical guidelines generally recommend initially choosing a lower dose of warfarin and closely monitoring the INR. Due to the difference in renal metabolism of different DOACs, apixaban has been reported to be safer and more effective than dabigatran and rivaroxaban (51). The effectiveness of apixaban is comparable to that of warfarin in patients with AF combined with severe CKD (CrCl <25 mL/min), and apixaban is safer than warfarin. In addition, the safety of apixaban is comparable to that of warfarin in patients with AF with ESRD (GFR <15 with dialysis). With increasing evidence-based support, apixaban has been recommended in updated guidelines for patients with AF who have severe CKD or ESRD, even for those undergoing dialysis.

## The Future of AF Anticoagulant Treatment

Factors that limit the long-term use of anticoagulants include the high bleeding risk associated with warfarin and the fact that the INR for effective anticoagulation overlaps with the risk of increased bleeding. Moreover, since AF patients tend to be older, have more comorbidities, and use more medications, it is more difficult to control the bleeding risk in these individuals. Compared with INR 2.0–2.9, the incidence of bleeding is twice as high with INR 3.0–4.4, four times as high with INR 4.5–6.0 and five times as high with INR >7.0 (52). Given this variation and because individual response to warfarin may be genetically determined, some researchers have started to explore the use of genetic testing to determine the therapeutic INR range upon initiation of warfarin therapy (53). In the case of emergent hemorrhage caused by DOACs, idarucizumab can quickly decrease the blood concentration of dabigatran to achieve rapid hemostasis. Alternatively, andexanet alfa, another antagonist of factor Xa inhibitors, is available for clinical use.

## Summary and Perspective

This review summarized the anticoagulant effect of warfarin and highlighted the advantages of DOACs for individuals with NVAF. However, the lack of effective monitoring, its price is more expensive than warfarin, and some anticoagulant antagonists are not even on the market in some areas, it is difficult to apply in large-scale clinical applications. Due to the lack of research on DOACs, the limited sample sizes of existing research may not fully reflect its advantages and disadvantages. Another important limitation of this paper is lack of meta-analysis and quantitative results.

The main advantages of DOACs include predictable pharmacokinetics, high efficacy, short half-life and rapid elimination of the effect after discontinuation, lower need for drug and dietary limitations, and lower intracranial hemorrhage risk without the need for frequent monitoring. With DOAC use, it is possible to improve patient compliance with long-term anticoagulant therapy, thereby increasing the treatment effectiveness rate for AF. The safety and effectiveness of DOACs need to be further verified by high-quality research data from more multi-center, double-blind RCTs (Randomized controlled trials).

## Author Contributions

XL designed the review. YZ and YL revised the manuscript. JW drafted the manuscript. All authors contributed to the article and approved the submitted version.

## Conflict of Interest

The authors declare that the research was conducted in the absence of any commercial or financial relationships that could be construed as a potential conflict of interest.
